# Undeserving, Disadvantaged, Disregarded: Three Viewpoints of Charity Food Aid Recipients in Finland

**DOI:** 10.3390/ijerph15122896

**Published:** 2018-12-17

**Authors:** Anna Sofia Salonen, Maria Ohisalo, Tuomo Laihiala

**Affiliations:** 1 Faculty of Social Sciences, University of Tampere, 33014 Tampere, Finland; tuomo.laihiala@uta.fi; 2 Y-Foundation, 00531 Helsinki, Finland; maria.ohisalo@ysaatio.fi

**Keywords:** food aid, charity, Finland, welfare state, food aid recipient, deservingness, disadvantages, inequality

## Abstract

Since the economic recession of the 1990s, Finland has experienced the proliferation of charity food aid as a means of helping people who are afflicted by poverty. However, so far little research has been conducted regarding the food aid recipients. This article gives discursive, demographic, and experiential insights into charity food provision and reception in Finland. Drawing on quantitative survey data, online discussion data related to news published on Finnish newspapers’ web pages, and observation and interviews with food aid recipients, this article sheds new light on Finnish food aid recipients from three perspectives. First, public perceptions about food aid often portray food recipients as dishonourable and responsible for their own poverty. Secondly, the survey data shows that the main reason for people resorting to charity food aid is deep economic disadvantage, and further, that there is an unequal accumulation of disadvantage among the food aid recipients, illustrating internal diversity. Third, observational and interview data show that from the food recipients’ perspective, the food aid system has only a limited ability to answer even their immediate food needs, and for the recipients, food aid venues can become not only socially significant, but also socially demanding and emotionally burdening places.

## 1. Introduction

Despite the almost thirty years of charitable food aid in Finland, so far little research has been conducted about the aid recipients. There have been a few studies examining the clientele of church diocese work and the food aid users at individual food banks [1,2,3,4,5]. This trend has changed only recently, as three studies have taken the initiative to explore Finnish charity food aid particularly from the users’ perspectives [6,7,8]. In this article, we use the existing data from these three studies to give a comprehensive picture of what is known so far about people receiving food aid in Finland.

Recent decades have witnessed the growth of food aid across the affluent world [9,10]. The global expansion of this phenomenon raises serious questions concerning food insecurity, public policy, and the future of welfare states. Food aid has prompted a lot of research in different parts of the world. However, there is still a need for more research on the various societal contexts in which food aid proliferates and on the viewpoints of the aid users [11] in terms of both who they are and how they perceive the aid they receive. With the concept of charity food aid, we refer to the phenomenon where non-governmental organizations (NGOs) provide free food to people who are living in poor social and economic situations; in contrast to statutory welfare provision, the food aid is voluntarily organized by the NGOs.

The Nordic welfare state context makes the Finnish case peculiar in relation to the many other countries where food aid has proliferated. In principle in Finland, the state is assumed to provide universal social security against social risks, such as poverty, for all its citizens. However, since the recession of the 1990s, Finland has experienced the proliferation of charity-based food aid provision as a means of helping people who are afflicted by poverty, indicating that the welfare state does not feed everybody. In Finland, food aid was initially considered a short-term response to the consequences of the recession of the 1990s, but it has gradually grown into an unorganized field, with hundreds of actors sharing food throughout the country. Over a quarter of a century, breadlines have become one of the most visible and well-known portrayals of poverty in Finland [12,13].

In the first cross-national study of charity food aid in the 1990s, it was stated that food aid is characteristic of residual welfare states, whereas the universalist Nordic welfare states have been able to safeguard social rights, such as the human right to food [14]. However, the Finnish case has challenged this perception. In her recent study comparing food aid and its implications for the welfare state in Finland and Scotland, Mary Anne MacLeod found that the rise of food assistance in Finland is coupled with the dilemmas of welfare state identity. Food poverty and food aid are considered marginal to the welfare state; food aid questions the effectiveness of the welfare system, and it is associated with societal failure. According to MacLeod’s study, in Finland, food is positioned as a public good, and thus charitable models of food aid provision are perceived as a threat to the social democratic welfare regime [15].

On the state level, it has been argued that the necessity for charity food aid contravenes the Finnish Constitution, which declares that everyone should have the right to a life of dignity guaranteed by the state. Section 19 in the Finnish constitution, ‘the right to social security’, explicitly lays the foundation for public social policy and social security, and points out the responsibility of the public authorities to safeguard social welfare and health. Finland has also signed the UN covenant on the Right to Food (RTF), which should guarantee freedom from hunger together with access to safe and nutritious food [9]. In other words, charity food aid raises particular disputes in the context of a Nordic welfare state that is presumed to guarantee basic social security for all its citizens.

Tellingly, food aid has even been called the ‘open wound’ of the welfare state [16] (p. 255). In public debates, it has been considered a deviant practice, since there should be no need for food aid in an affluent Finnish society with a comprehensive social security system. At the same time, the efforts of churches and NGOs to provide food aid have been applauded. The perception of food aid thus holds an ambivalent position in Finnish public discourse: charitable food assistance is not fitting for the Nordic welfare state, but it is an appropriate way for churches to help the needy [17]. Thus, Finland marks an interesting case where the strong constitutional responsibilities of the state meet widespread unofficial aid provided by a lively and diverse non-governmental sector.

Due to this particular discrepancy between the strong welfare state ideal and strong grassroots charity aid, the connection between professional social work and food aid is in principle absent in Finland. There is no referral system between charity food providers and social services, and it has even been considered unconstitutional for social workers to inform or guide their clients to charity food aid services [18,19]. In other words, there are no explicit connections between food aid and public social policy. Illustrative of this gap on the state level is the fact that the administration of the EU’s food aid programme in Finland was first set up under the Ministry of Agriculture and Forestry—and later the Ministry of Employment and the Economy—instead of the Ministry of Social Affairs and Health ([13], p. 476). Interestingly, however, many of the non-governmental organizations providing food aid receive some public funding—from local municipalities, for example—to support their non-profit work. Nevertheless, this funding is not targeted at food aid per se, but to the infrastructures and general activities of the organizations. In practice, then, food aid is often publicly supported, though only partly and indirectly.

On the grassroots level, the characteristic features of Finnish charity food aid are a low-level of organization and a lack of eligibility control. Unlike in many other countries, there are hardly any intermediaries in Finland that could collect and store food and redeliver it to local charities. Instead, local actors most often collect, store, and redistribute the food independently and according to their own individual practices [20]. The methods of providing assistance vary across the individual food aid organizations, but very often food aid provision is based on the principles of low threshold and the absence of means tests. Some food aid providers might ask to see proof of the recipient’s status as unemployed or a pensioner, for example, but a detailed income assessment is rarely conducted. The basic principle is that asking for food aid is in itself a sign that the recipient deserves the aid. Thus, in many assistance venues, technically anyone can ask for and receive charity food aid.

Due to a lack of coordination, shared practices, or comparable statistics, only rough approximations can be drawn about the volume of food aid in Finland. A 2013 survey estimated that food assistance was available in over 220 of the more than 300 municipalities throughout the country [20]. The food aid is distributed via various faith-based and other NGOs. The food comes from two main sources: the EU food aid programme and food companies and grocery stores donating their surplus food. In addition, public institutions such as schools have recently started to give out surplus meals to charities. Based on the assessment of food aid distributors, approximately 20,000 people received food aid rather regularly in 2013 [20]. However, a national-level survey asking whether respondents had used food aid at least once a year found that more than four times that number had received food aid [21]. The Evangelical-Lutheran Church in Finland gave food in the form of free or cheap meals or food packages to roughly 56,000 people in 2015 [22]. These are significant figures in a country with a population of approximately 5.5 million people. For comparison, in 2015, 634,000 people, or 11.7% of the Finnish population, were considered low-income—that is, they belonged to the population living on less than 60% of the equivalent median money income of all households [23].

Overall, the Finnish food aid system can be described as an unorganized yet widespread practice of unofficial, last-resort aid targeted at people living in difficult social and economic situations. Moreover, the system has no strict criteria for eligibility. This peculiar situation raises many questions. First of all, the lack of objective criteria for food eligibility provokes a normative debate concerning deservingness—that is, who should get what, and why [24,25]. Who should be granted the moral entitlement to use assistance that is in principle available to everyone, but which is at the same time contrary to the Finnish welfare ethos? Second, the situation raises a policy question concerning the populations involved in this widespread yet abnormal form of aid. In the absence of guidelines and practices shared between different food aid providers, it is very hard to estimate who the food assistance recipients are or to determine their reasons for using food aid. Third, such an unregulated and unofficial setting calls for an exploration of the experiences of the recipients. What are the repercussions of food aid use for these individuals? Without research addressing these questions, preconceptions flourish and colour the public and policy discussions on the issue.

In this article, we examine the Finnish charity food aid recipients from three distinct perspectives. First, we present findings from a study that analyses the online perceptions of food aid recipients to illustrate the discursive landscape in which Finnish charity food aid is rooted. Second, drawing on quantitative survey data collected among food aid recipients, we bring new light to the often-held assumptions about who the food aid recipients actually are. Third, we use observation and interview material from Finnish food banks to illustrate how the aid is experienced by the recipients. By bringing these findings together, we aim to provide a holistic picture of the food aid recipients in Finland. Together, the findings presented in this article provide discursive, demographic, and experiential insights into charity food provision and reception in the Finnish context, thus giving a novel account of charity food aid in an affluent, Nordic welfare state from the viewpoint of the people whom this aid concerns the most.

## 2. Materials and Methods

In this article, we present findings from recently conducted studies that utilize data from three sources. First, we present findings from online discussion data related to news published on Finnish newspapers’ web pages to understand how the food aid recipients are perceived in public discourses. The data consist of 1294 comments collected from online discussions that were connected to news articles about food aid in nine prominent Finnish newspapers (*Aamulehti*, *Helsingin Sanomat*, *Iltalehti*, *Ilta*-*Sanomat*, *Länsiväylä*, *Metro*, *Satakunnan Kansa*, *Taloussanomat*, *Turun Sanomat*) in 2014 and 2015. The data were analysed with close reading, and a topic model was created with GUI Topic Modelling -programme to cover all the relevant themes. The themes that occurred in the data were interpreted in the light of Wim van Oorschot’s criteria for deservingness, including need (the greater the level of need, the more deserving), control (poor people’s control over their neediness, or their responsibility for it), identity (the identity of the poor, i.e., their proximity to the rich or their “pleasantness”), attitude (poor people’s attitude towards support, or their docility or gratefulness), and reciprocity (the degree of reciprocation by the poor, or having earned support) [25]. The data collection and analysis is described in detail in [6].

Second, we present data from a quantitative survey that researched both the socio-economic status of food aid recipients and the accumulation of the recipients’ disadvantages (see the Supplementary Materials for the English version of the survey form). This is the first and so far only study where the socio-economic position and disadvantages of the Finnish aid recipients has been studied with larger-scale survey data. The data were collected in a national food aid study (*N* = 3474) in 2012–2013 from 37 different charity food aid distributions in 11 Finnish municipalities. The food aid venues chosen for this study were known to be the largest in Finland in terms of the number of food aid recipients. As the number of people receiving food aid in Finland is unknown, the demographic sample does not necessarily represent all the food aid recipients in the country. However, the results from different municipalities are relatively uniform, indicating that the data sample captures a good overall picture of the food recipients. Surveys were distributed in three different languages—Finnish, Russian, and English—and the researchers who collected the surveys helped the respondents in translating them according to the situation. The study targeted the subjective well-being of the food aid recipients. The data were analysed with SPSS (IBM Corporation, Armonk, NY, USA) using multivariate methods, namely factor analysis, cluster analysis, and cross tabulations. The data collection and analysis is described in detail in [7].

Third, we present findings from a qualitative study that consist of observational notes from over seven months of participant observation in four food assistance organizations, written documents related to the operation of the organizations, and open-ended interviews with 25 food aid recipients. The data were collected from four food charity organizations in the city of Tampere, Finland, in 2012 and 2013. The selection of one of the large cities in Finland enabled the researchers to uncover possible variations in the different kinds of food aid venues and to reach a wider group of food recipients. The data were analysed with qualitative methods, such as qualitative inductive content analysis and grounded theory, where conceptions of different incidents, venues, people, and occasions were constructed and compared in order to develop a comprehensive understanding of the phenomenon. The data collection and analysis is described in detail in [8]. In this article, we discuss the findings that relate to the ability of food aid to meet the needs of the recipients.

**Figure 1:**
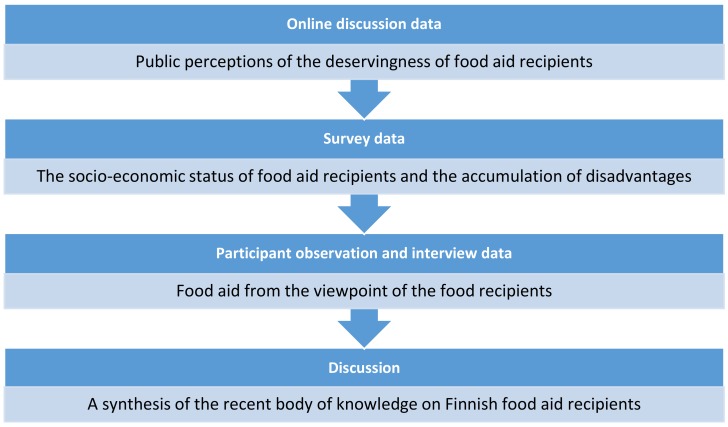
The outline of the study.

In the subsequent sections, we first present the findings from these different data sets and then draw a synthesis of this recent body of knowledge on Finnish food aid recipients: we discuss how they are perceived by the public, who they actually are, and how they themselves see their own social position and the phenomenon they are engaged in (Figure 1). In the discussion section, we discuss these combined findings to show how they raise some significant issues regarding food aid recipients.

## 3. Results

### 3.1. Public Perceptions of Food Aid Recipients’ Deservingness in Online Discussions

The online discussion data shows that Finnish food aid recipients are exposed to strong public criticism and blame. Of the themes covered in the discussions, the most prominent was the issue of need: the discussants questioned whether the food recipients were in need of food aid, for example, by suggesting that the recipients squander their money and then request assistance. The emphasis on need is surprising given that the needs-based arguments of deservingness do not fit well with the Finnish welfare state context.

The analysis of the online discussion data shows that the discussants differed based on how they related to the need of the food aid recipients and how they perceived the causes and reasons for the food aid use. The discussants who considered the food aid recipients to be in genuine need expressed their desire to help and give support and encouragement to the disadvantaged. Those who acknowledged the need but also blamed the recipients for their situation considered obtaining charity food aid acceptable only if the recipients were genuinely in need of help. However, the needs and motives of most of the recipients were questioned, and they were presumed to be caused by lifestyle choices. Furthermore, some of the online discussants maintained that food aid represents a systemic problem: in a good society, charity food aid should not be needed. The poor life situation of an individual is a matter for society and the welfare state rather than the fault of the individual. Finally, some of the discussants questioned the food recipients’ need and pigeonholed them as undeserving scroungers.

Another central topic that surfaced in the discussions was the question of who is responsible for poverty. Unlike in previous quantitative research that found Finnish people tend to see poverty as a structural problem [26,27], a significant number of the online discussants considered the situation of the food aid recipients to be self-inflicted. The recipients’ need was often questioned, and the recipients were considered a dishonourable group responsible for their own poverty. In its considerable resemblance to traditional aid for the poor, Finnish charity food aid enables this kind of discussion about deservingness, which fits poorly with an institutional welfare state.

Not all online discussants condemned the charity food aid recipients. Some defended the recipients’ deservingness and considered them unfortunate, disadvantaged people who have to rely on charity food as a result of society’s failures. Empathy, solidarity, and positive attitudes towards the recipients can be predicted by the discussant’s personal or other close experiences with charity food aid and economic disadvantage in general. The analysis found that the discussants questioned the deservingness of the food aid recipients and emphasized their own responsibility particularly when the food aid recipients were not considered to belong to the same social group as them. The most conditional were the attitudes towards immigrant food recipients.

Unlike in studies that found gratitude and shame to be the prominent emotions expected of the food aid recipients [28], the Finnish online discussants rarely required the food recipients to perform emotional or attitude-related responses towards the aid or the aid providers. Instead, the food aid itself was seen by the discussants as humiliating, either for the food recipient or from the perspective of wider society.

### 3.2. The Socio-Economic Status of Food Aid Recipients and the Accumulation of the Recipients’ Disadvantages

Perceptions of the extent of food aid in Finland, the position of aid recipients in the social security system, and their usage of services and benefits are often based on impressions rather than on systematic, empirical information. According to many food aid distributors, the picture of food aid recipients has diversified since the recession of the 1990s. Previously, it was often unemployed or homeless men queuing for food, but nowadays the charity food aid venues bring together people from a variety of backgrounds. The findings of the national food aid study presented here provide empirical evidence of the recipients’ socio-economic position and disadvantages.

The socio-economic status of people receiving food aid was outlined with 11 questions. To begin with age, the biggest age group of food aid recipients was 46–65-year-olds. Young people tend not to be highly represented in Finnish food aid venues. There are several reasons for this; for example, students receive subsidized meals at the university level, and many of them complement their income by working part-time during their studies. Thus, the people receiving food aid seem to be older compared to the demographic structure of Finland in general (see Appendix A for the results compared to the general population of Finland).

Unlike in many other disadvantaged groups, the gender division among food aid recipients was nearly non-existent. There was only a small majority of women (51.7%, *N* = 1704) receiving food aid, even though men tend to be overrepresented in many disadvantaged groups. The majority of the people receiving food aid were native Finns (87.3%, *N* = 2817).

One stereotype about people receiving food aid in Finland is their assumed low educational background. However, the data partly challenge this supposition. In the food aid venues, there were more people with only a basic level of education (39.6%, *N* = 1270) and fewer people with a higher education background (20.4%, *N* = 656) compared to the general population in Finland. Nevertheless, the relative amount of the people with an upper secondary level education (40%, *N* = 1282) was nearly the same as it is among the wider Finnish population.

In terms of employment status, food aid recipients were characterized by a weak labour market position. Roughly four fifths of them were either pensioners (38.4%, *N* = 1260) or unemployed or laid off (38.4%, *N* = 1260). One in seven respondents were at home (7.3%, *N* = 240) or students (6.6%, *N* = 215). Many of the student respondents were working while studying, but their main occupation was recorded as ‘student’. The phenomenon of the working poor is seen in food aid, as one in ten food aid recipients were people working part-time or on a fixed-term contract (5.6%, *N* = 185), or full-time (3.7%, *N* = 120).

In terms of housing, the majority of the respondents (78%, *N* = 2570) lived in a rented property, and only 16% (*N* = 527) owned their own home. On the national level in 2011, the percentages were nearly the reverse: 59% lived in owner-occupied dwellings, whereas only 29% of the people lived in rented dwellings. Homeless respondents (3.3%, *N* = 109) and people living in supported housing (2.8%, *N* = 92) were a small minority. However, these figures exceed the national levels, as roughly 8000 (0.15%) people in Finland were homeless at that time. The size of the household was measured by asking the number of adults and children living in the household. Of the respondents, over three fifths (60.5%, *N* = 2024) lived alone, whereas on the national level two fifths live in one-adult households [29].

In terms of the frequency of food aid use, nearly one third (*N* = 952) of the food aid recipients obtained charity food weekly. One quarter (25.9%, *N* = 816) received food aid approximately every two weeks, and one fifth (20.1%, *N* = 633) received food aid roughly once a month. Under a quarter (23.9%, *N* = 752) of the respondents received food aid only couple of times a year. A majority of the recipients of the food aid got the food for themselves (47.6%, *N* = 1544), but over two fifths (42.6 %, *N* = 1380) picked up food for themselves and their families. One in ten (9.8%, *N* = 317) got the food for themselves and other non-family members.

In terms of the money left over after each month’s compulsory outgoings, the results show that nearly half of the respondents (44.5%, *N* = 1316) were left with 0–100 euros. One third (30.9%, *N* = 913) had 101–300 euros, and a quarter (24.7%, *N* = 730) had more than 301 euros per month.

It is known from Finnish national-level surveys that disadvantages tend to accumulate in three main dimensions: economic, social, and health [30]. When researching the disadvantages of the respondents, the findings show that the same dimensions found in studies representing the Finnish population were also found among the food aid recipients (Table 1). The results are statistically significant. One quarter of the respondents had not experienced severe economic disadvantage or accumulated disadvantages, although they were less well off when compared with the wider population. Typically, people belonging to this group were pensioners and the working poor living on social assistance or a guarantee pension and experiencing high levels of scarcity. Most of the people (three quarters) receiving food aid had deep economic problems, such as difficulties in making ends meet and paying debts. These were mainly young people, students, and people with families.

Notably, over two fifths of the people receiving charity food aid suffered from several simultaneous disadvantages. They not only had problems with their economic situation but also health disadvantages, such as poor mental and/or physical health and lower levels of life satisfaction. In addition, they experienced social disadvantages such as hunger, loneliness, and depression. In this group, the homeless, unemployed, substance abusers, and people with the least disposable income were overrepresented.

Overall, based on the data, people receiving food aid in Finland are a heterogeneous group. However, the group has a poorer employment status compared to the wider Finnish population, and is older, less educated, and on a lower income. People receiving food aid mostly suffer from economic deprivation. They are also more likely to live alone. Moreover, two fifths of the food aid recipients live with accumulated economic, social, and health disadvantages.

### 3.3. The Food Recipients’ Viewpoint of Food Aid

The sections above illustrate that while the public perception of food aid recipients mostly presents these people as a homogeneous group, in reality food aid recipients come from various walks of life, and they experience disadvantages of various degrees and intensities. What, then, do these people themselves think about the assistance they receive? The qualitative data on the food aid recipients’ perspectives of the assistance further complement the above findings. As in the survey data, the informants of the qualitative study were a heterogeneous group that came from various backgrounds. The common denominator for the informants was a low income and the concomitant need for material assistance. For these recipients, using food aid was a practical coping mechanism for dealing with a weak social and economic situation; it was relief that helped in managing everyday scarcity.

However, even though food aid alleviates the immediate food needs of the recipients, the study found out that there are limitations in the food aid system’s ability to satisfy these needs. The finding is in line with previous research that suggests food aid does not address the root causes or structural problems behind food insecurity [31,32,33]. Furthermore, the study found that the food aid system has only a limited ability to meet the immediate food needs of the recipients. This was particularly the case due to the detachment of the food resources in the food assistance venues under study from the needs of the food recipients. Much of the food delivered to these venues was market surplus, and thus its quality and quantity was dependent on what happened to be left over from the primary food markets. Moreover, some of the venues also redistributed food from the EU’s food programme, which did not completely align with the needs of the food recipients.

There were problems regarding both the amounts of food available and the quality of food: even though there was occasionally plenty of food available, the food recipients had difficulties in utilizing it. Thus, the occasional abundance of food highlights the inconsistency between the food needs and the food supply in the food aid venues. In terms of the material needs of the food recipients, food aid seems to be able to alleviate only the direct, immediate food needs of these people, and even those only insofar as the needs correspond with what happens to be available.

In addition to their food needs, many informants mentioned social reasons for coming to food aid venues, such as meeting other people, spending time, and enjoying the additional social and religious programmes that some of the food aid providers integrated in the food delivery events. This finding is in line with the quantitative survey study, which found that 53% of the respondents agreed with the statement that it is important for them to meet other people in the food aid venues [34].

Recently, the communal and social aspect of food assistance has gained prominence in Finnish public discussions about food assistance. There are efforts to remodel food aid to provide the participants with communal experiences. However, the findings of this study reveal that from the perspective of the food aid recipients, the communal and social aspect of food aid is not only a positive feature. Occasionally, the low threshold and lack of eligibility control that aimed at inclusiveness resulted in adverse outcomes, such as mutual surveillance among participants and both subtle and hash negotiations over who should receive food first. Thus, the study highlights that food banks are communities with various communal qualities, and not all of them are positive. For the recipients, food aid venues can be socially significant yet socially demanding and emotionally burdening places. From the perspective of the recipients, it is thus important to acknowledge that these venues are about ‘more than bread’—both in the good, and in the bad.

In addition to the material and social challenges faced by the food aid recipients, the study found that the informants encountered restrictions on their ability to express their needs, expectations, and experiences related to the assistance. For example, the recipients could only subtly express criticism towards the quality or practical usability of the food items, even in matters regarding food safety. To give an example, one informant delicately noted when he realized that the expiry date of a food item had passed a while ago: ‘I really don’t dare to eat those meat products. I am not picky, but…’ In the context of food aid, the exercise of consumer choice was restricted and even resented. In everyday discussions, criticism was aimed at individuals who were considered choosy. One interviewee remarked aptly how, in the food aid context, ‘[y]ou have to be something like a piggy. You eat what comes. Yes. […] If you choose, you starve!’ The interviewee thus hints that in a food bank, exercising choice regarding food might lead to receiving nothing. As a further example, one recipient lamented how ‘there are those finicky ones, who [do not eat particular food stuffs even though they] are not allergic, or anything. But if they can afford to...’ Implicit in this statement is the idea that food aid recipients do not have the right to choose the content of the aid. As the latter part of the comment suggests, refusing certain food items indicates that one is not really in need of aid, which hints at the discussions of deservingness presented above.

The recipients’ limited choices were also present in their limited ability to withdraw from food aid use. This became apparent in situations where the informants spoke about the social and emotional stress that food aid use caused for them. For example, one recipient stated, ‘I feel that it would be easier not to come. But how do I cope then? Where do I get [food] then? I don’t know where I would then get [food], and I don’t know what I should do. But it is like, it is already quite depressing.’ The restricted agency of the food recipients means that due to their harsh economic situations, they rarely have a chance to decide whether or not make use of the assistance food without tremendous disadvantages. However, they rarely have the ability to express their needs, outlooks, and feelings related to the assistance, either. In many ways, their needs and aspirations remain overlooked.

## 4. Discussion

The above findings shed new light on the recipients of Finnish food aid from various perspectives. First, public perceptions of food aid in the online discussions often portray the recipients as dishonourable and responsible for their own poverty. At the same time, the quantitative data reveal that the main reason for people resorting to charity food aid is deep economic disadvantage. Furthermore, the quantitative data show that there is an unequal accumulation of disadvantage, illustrating the internal diversity within food aid recipients. Finally, observational and interview data show that from the food recipients’ perspective, food aid provision disregards the material and social needs of the food recipients. The assistance system has only a limited capacity to meet even the recipients’ immediate food needs, and for the food assistance recipients, food aid venues can become not only socially significant, but also socially demanding and emotionally burdening places.

Together, these findings point out some significant issues regarding food aid recipients. First, the findings from the online discussions indicate that from perspective of outsiders, the food aid recipients are often seen as a homogeneous group, alien to the majority population. Paradoxically, the life situations of the food aid recipients are often evaluated by arguing that there should not be severe poverty in a Finnish welfare state. As a result, if and when one is afflicted by poverty, the need for help is questioned and the individual is blamed [35]. Hence, it is important to gather empirical data to understand who the food recipients really are and what their socio-economic status is. The survey data of the food aid recipients bring facts to the public discussion, where the stereotypical picture of a food aid recipient is an uneducated, poor, typically male substance abuser standing in a breadline. The data can reveal the inner diversity of this group and the fact that many of the recipients are living in weak social and economic positions when compared to the wider Finnish population.

Second, the quantitative data reveal that food aid recipients suffer from economic, social, and health disadvantages. In addition, qualitative data show that they suffer from disadvantages in the form of social exclusion from the consumer practices of the wider population. Further, the findings indicate the inner polarization among food aid recipients: the survey data show that there is an accumulation of disadvantages in certain groups, while the qualitative analysis of the experiences of the food recipients highlight experiences of social exclusion and being left without.

Finally, on the level of public perceptions, food aid recipients are judged based on their perceived deservingness. However, at the same time, from the perspective of the food recipients the question arises of whether the available food aid meets their needs in the first place. The study of the food aid from the perspective of the Finnish food assistance recipients highlights the ambivalent social position that the recipients hold. First of all, they are excluded from ways of acquiring food that are customary in contemporary society. At the same time, they are dependent on the consumer practices of the affluent population that secure the continuous flow of excess. Second, socialization into the food aid community might promote the institutionalization of food aid on the individual level and entrench the food aid recipients’ social exclusion from wider society. Food aid serves as an instrument for polarization that distances the life worlds of the disadvantaged people and the well-off majority. As seen in the findings from the online discussions, the public perceptions of food aid recipients feed back into these experiences and aggravate the social divide.

## 5. Conclusions

There are certain limitations to this study that should be taken into account when interpreting the findings. First of all, the data used in this article were collected some years ago already, and thus they do not present the most recent situation. From a research perspective, it is unfortunate that there are no up-to-date data readily available. On the other hand, this situation well illustrates the ad hoc and unorganized field of food aid in Finland. There are no registers or any other reliable data available about food aid recipients in Finland. Charity food aid recipients comprise one of the so-called hard-to-survey populations [36]: people receiving food aid tend to be hard to find or contact, as they are not found via post or phone surveys; they are occasionally difficult to persuade to participate, as going to food aid is stigmatizing for many; and being anonymous is important for some [37]. Furthermore, they can be difficult to interview, as there is not always a common language, some might be illiterate, some might be intoxicated, and some might be generally reluctant to take part in research. These are only some of the difficulties faced in interviewing food aid recipients. We have relied on data from 2012–2013, because they represent the first and so far only consistent quantitative information about the Finnish food aid users.

Second, it is worth noting that the data from the online discussions about food aid is not representative when it comes to the general populations’ perceptions and attitudes. About 80% of Finns follow online media sources. Still, relatively few readers use the opportunity to comment on and discuss the news online. Strong opinions and active debaters gain the most visibility online [38]. However, keeping this limitation in mind, the online discussions provide an interesting viewpoint to approach public perceptions about food aid, because they offer—albeit in aggravated form—an indication of the traits that represent the general public’s attitudes. Furthermore, the mindsets expressed online have the potential to spread outside the online debates, and thus it is helpful to be aware of them.

Third, since this article uses existing sources of data that have been each collected for the particular purposes of the original studies, one should be cautious when discussing the combined findings. In this article, we have settled on discussing the connections between different data sets descriptively instead of conducting cross-data analyses about each domain of the results. The discussion of the findings shows that different data sources complement each other, and together they help to paint a more nuanced picture of the reality in which food aid takes place in Finland and where the food aid recipients make do. Further research is needed that more thoroughly integrates discursive, demographic, and experiential insights into charity food provision and reception.

Despite these limitations, the study offers valuable insights into Finnish food aid, as it brings together the current body of knowledge about Finnish food aid recipients. In doing so, it shows that unlike in other Nordic countries such as Norway and Sweden, where the food aid clientele often represents the very margins of society [39,40], the recipients in Finland make up a relatively wide and diverse group. The food aid recipients are more typically of an older age, lower education, and lower income compared to the wider Finnish population. They are also more likely to have a weaker employment status and live in a one-person household. At the same time as the public debates about their deservingness, the food recipients themselves suffer from (often accumulated) economic, social, and health disadvantages. For them, the aid is important—if not necessary—to cope in their everyday life, even though they simultaneously struggle to make use of the aid, which does not always fall in line with their wants and needs.

Despite the general image of Finland as an affluent welfare state, there are tens of thousands of people who need to resort to charitable food aid in order to cope in their everyday lives. In February 2018, the annual fundraising campaign of the Finnish Lutheran Church, called the ‘Common Responsibility Campaign’ (*Yhteisvastuukeräys*), launched its annual campaign with the theme of hunger and poverty. With the domestic part of the proceeds, the campaign aims to provide one-off subsidies and food aid for low-income households in Finland. With its poignant hashtag #foodtrends, the campaign underlies the ambiguity of today’s Finnish society where some people feast while others fast or starve [41]. This example illustrates that the issue of food aid is far from diminishing in Finnish society. Rather, it is becoming institutionalized, and it is gaining public recognition. One distinguishing feature of Finnish food aid has been the relative absence of a charitable culture attached to the aid; this is in contrast to the United States and Canada, for example, where private individuals and corporations are invited and encouraged to donate food for charitable purposes through prominent popular campaigns [42,43]. In the future, the proliferation of visible ‘hunger campaigns’ in Finland might influence who receives food aid, how the aid is experienced, and how its recipients are perceived by the public. More research is needed about food aid recipients in this changing landscape.

With charity food aid, the issues of poverty and food insecurity have been shifted to the margins and the purview of NGOs and third-sector voluntary aid. However, it is ideally a public responsibility to take care of people who experience poverty. Leaving the responsibility for the care of this vulnerable group to voluntary and religious actors indicates a neglect of the constitutional and basic rights of these people, especially the right to food [10,13].

Poor relief is always stigmatizing. People who receive last-resort charitable aid are exposed to public judgement, which is likely to weaken their well-being. Charity food aid also provokes discourses of deservingness that are alien to the universalist welfare model [24]. In the light of the findings, there is a legitimate need for assistance. However, this need cannot be met solely by giving people food as charity. Rather than deservingness, the focus of public concern ought to be on how the official social security system could be developed so that it can respond to the life situations of those who are afflicted by poverty. Mapping the actual needs and reasons for food aid use requires more research knowledge on the life worlds of the people who live in vulnerable social and economic positions.

Finally, charitable food aid venues are often one of the only places where the most deprived members of society can be found. This fact could be used as an asset when planning more effective ways to tackle poverty and food insecurity. Information and research knowledge about food aid in general, and food aid recipients’ wellbeing and experiences in particular, should be systematically gathered and made available in order to alleviate poverty and food insecurity more effectively.

## Figures and Tables

**Table 1:** Accumulation of disadvantages and people affected by economic, social, and health disadvantages.

**How Do Disadvantages Accumulate?**	Less well-off compared to the wider population, no accumulated disadvantage, 24.7% (*N* = 693)	Severe economic disadvantage (without other disadvantages), 33.7% (*N* = 945)	Strongly accumulated economic, social, and health disadvantage, 41.5% (*N* = 1163)
**What does it mean?**	Does not suffer from severe economic or accumulated disadvantage	Suffers from severe economic disadvantage, but not from social or health disadvantages; has difficulties in making ends meet and paying debts; is dissatisfied with the current standard of living and has experiences of insufficient support	Severe economic disadvantages; disadvantages in mental and physical health and lower levels of life satisfaction; social disadvantages such as hunger, loneliness, and depression
**Who is affected?**	Pensioners and the working poor living on social assistance or a guarantee pension and experiencing high levels of scarcity	Young people, students, and people with families	The homeless and people living in supported housing, the unemployed and laid-off, substance abusers, people considering themselves disadvantaged, people with the least money to spend freely, and people using last-resort social support

## Supplementary Materials

The following are available online at http://www.mdpi.com/1660-4601/15/12/2896/s1, S1: research on food aid 2012.

## Appendix A

**Table A1:** Socio-economic background of food aid recipients compared to the general population in Finland (2012–2013).

	Food aid recipients		General population of Finland
	N	%	%
Age (in full years)			
16–25	199	6	12.2
26–35	356	10.7	12.6
36–45	512	15.4	12.1
46–55	789	23.7	13.7
56–65	893	26.9	14.2
Over 65	574	17.3	18.8
Gender			
Male	1592	48.3	49.1
Female	1704	51.7	50.9
Nationality			
Finnish	2817	87.3	96.4
Other	410	12.7	3.6
Education			
Comprehensive school	1270	39.6	32
Upper secondary school/Vocational school	1282	40	40
University	656	20.4	28
Employment status			
At home	240	7.3	
Pensioner	1260	38.4	
Unemployed or laid off	1260	38.4	
Student	215	6.6	
Working fixed term or part-time	185	5.6	
Working under permanent contract	120	3.7	
Housing			
Home owner	527	16	59
Rental accommodation	2162	65.6	29.1
Council accommodation	408	12.4	
Supported living	408	2.8	
Homeless	109	3.3	0.15
Recipient of food aid during the last year			
A few times a year	752	23.9	-
Approximately once a month	633	20.1	-
Approximately every other week	816	25.9	-
Every week	952	30.2	-
Getting food			
Only for myself	1544	47.6	-
For myself and my family	1380	42.6	-
For myself and others	317	9.8	-
Number of adults in a household			
1	2024	60.5	41
2 or more	1324	39.5	59
Number of children in a household			
0	2403	71.6	
1	412	12.3	
2 or more	543	16.2	
Money (€) left after each month’s compulsory outgoings			
0	607	20.5	
1–100	709	24	
101–300	913	30.9	
301–500	429	14.5	
Over 500	301	10.2	
