# Supplementary material for: Undeserving, Disadvantaged, Disregarded: Three Viewpoints of Charity Food Aid Recipients in Finland
Source: Int J Environ Res Public Health. 2018 Dec 17;15(12):2896. doi: 10.3390/ijerph15122896 (PMC6314006; doi:10.3390/ijerph15122896)
Supplement: Supplementary file 1 [file ijerph-15-02896-s001.pdf]

Research on food aid 2012

Please answer the following questions by choosing the option that best describes your situation or experiences. No personal information will be collected.

1. **When you think about your life over the last month, have you felt**

	Constantly	Quite often	Sometimes	Very rarely
Satisfied with your life				
Satisfied with your standard of living				
Satisfied with your physical health				
Satisfied with your mental health				
Depressed				
Lonely				
Hungry				

2. **From the following statements please choose the option that best describes your view.**

	Fully agree	Partly agree	Neither agree nor disagree	Partly disagree	Fully disagree
I feel disadvantaged.					
I cope in my life.					
People can be trusted.					
Authorities can be trusted.					
My income is enough to cover my outgoings.					
I can manage my debts.					
I get enough support, help and services from the municipality.					
I get enough support, help and services from the parish.					
In the future my life will be better.					
I get financial aid from my friends and family if needed.					
I have problems with substances (like alcohol or drugs).					

3. **Which of the following benefits and subsidies provided by the municipality, Kela (the Social Insurance Institution), pension institutions have you or any member of your family received during the last two months?**

	I have received	I have applied but not received	I have not applied
Income support			
Student grant			
Housing allowance			
Disability allowance			
Sickness allowance			
Maternity/Paternity allowance			
Child home care allowance			
Unemployment benefit			
Earning related unemployment allowance			
State pension			
Disablement pension			
Unemployment pension			
Old-age pension			

4. **Which of the following services provided by the municipality, parish and Kela have you used during the last two months?**

	I have used	I have applied but have not received	I have not used
Mental health services			
Substance abuse services			
Social services			
Health care services			
Kela services (rehabilitation, therapy, etc.)			
Parish services (deacobess services)			
Unemployment benefit services			

5. **Please choose the option that best describes your views about food aid.**

	**Fully agree**	**Partly agree**	**Neither agree nor disagree**	**Partly disagree**	**Fully disagree**
**Food aid is essential for my survival.**					
**Getting food aid is humiliating to me.**					
**Food aid is well organised.**					
**I don't want my neighbours or relatives to see me getting food aid.**					
**In the queue there are people who do not need the food provided.**					
**The food given is of good quality.**					
**Meeting other people in the queue is important to me.**					

6. **Please circle the most suitable options that relates to your background.**

**Gender**	**Male**	**Female**				
**Nationality**	**Finnish**	**Other**				
**Age (in full years)**	**16-25**	**26-35**	**36-45**	**46-55**	**56-65**	**over 65**
**Education**	**Comprehensive school**	**Upper secondary school/Vocational school**	**University**			
**Employment status**	**Working permanently**	**Working fixed term or part-time**	**Unemployed or laid off**	**Student**	**At home**	**Pensioner**
**Housing**	**House owner**	**Rental accommodation**	**Council accommodation**	**Supported living**	**Homeless**	
**Customer of the food aid during the last year**	**Every week**	**Approximately every other week**	**Approximately once a month**	**A few times a year**		
**I also visit other food aids**	**No**	**Every week**	**Approximately every other week**	**Approximately once a month**	**A few times a year**	
**Getting food**	**Only for myself**	**For myself and my family**	**For myself and others**			

7. **Please answer the following in numbers.**

**Members of the household**	**Number of adults:___________**	**Number of children:___________**
**After each month's compulsory outgoings (housing, food, etc.), how much money have you got left (nearest 100 €) :___________€**		

Thank you for your answer!
